# Intergenerational transmission of complex traits and the offspring methylome

**DOI:** 10.1038/s41380-025-02981-7

**Published:** 2025-04-03

**Authors:** Fiona A. Hagenbeek, René Pool, Austin J. Van Asselt, Erik A. Ehli, August B. Smit, Meike Bartels, Jouke Jan Hottenga, Conor V. Dolan, Jenny van Dongen, Dorret I. Boomsma

**Affiliations:** 1 Department of Biological Psychology, Vrije Universiteit Amsterdam, Amsterdam, The Netherlands; 2 Amsterdam Public Health (APH) research institute, Amsterdam, The Netherlands; 3 Institute for Molecular Medicine Finland (FIMM), HiLIFE, University of Helsinki, Helsinki, Finland; 4 Avera McKennan Hospital, University Health Center, Sioux Falls, SD USA; 5 Department of Molecular and Cellular Neurobiology, Center for Neurogenomics and Cognitive Research, Amsterdam Neuroscience, Vrije Universiteit Amsterdam, Amsterdam, The Netherlands; 6 Neurological Disorder Research Center, Qatar Biomedical Research Institute (QBRI), Hamad Bin Khalifa University (HBKU), Qatar Foundation, Doha, Qatar; 7 Amsterdam Reproduction & Development (AR&D) research institute, Amsterdam, The Netherlands; 8 Department of Complex Trait Genetics, Center for Neurogenomics and Cognitive Research, Amsterdam Neuroscience, Vrije Universiteit Amsterdam, Amsterdam, The Netherlands

**Keywords:** Genetics, Schizophrenia

## Abstract

The genetic makeup of parents can directly or indirectly affect their offspring phenome through genetic transmission or via the environment that is influenced by parental heritable traits. Our understanding of the mechanisms by which indirect genetic effects operate is limited. Here, we hypothesize that one mechanism is via the offspring methylome. To test this hypothesis, polygenic scores (PGSs) for schizophrenia, smoking initiation, educational attainment (EA), social deprivation, body mass index (BMI), and height were analyzed in a cohort of 1528 offspring and their parents (51.5% boys, mean [*SD*] age = 10 [2.8] years). We modelled parent and offspring PGSs on offspring buccal-DNA methylation, accounting for the own PGS of offspring, and found significant associations between parental PGSs for schizophrenia, EA, BMI, and height, and offspring buccal methylation sites, comprising 16, 2, 1, and 6 sites, respectively (alpha = 2.7 × 10^−5^). More DNA methylation sites were associated with maternal than paternal PGSs, possibly reflecting the maternal pre- and periconceptional environment or stronger maternal involvement in shaping the offspring’s environment during early childhood.

## Introduction

Phenotypic resemblance among parents and their biological offspring can be due to both genetic and environmental influences, with the genetic contribution to phenotypic resemblance manifesting through different sources. The first source applies to all heritable phenotypes: the Mendelian transmission of parental alleles to their offspring, resulting in parents and offspring sharing 50% of their autosomal alleles. A second source is heritable parental behaviors, which shape the environment of the offspring. Here, the effect of parental genotype on offspring phenotypes is mediated by parental traits and behavior, contributing to the offspring environment. This source is denoted “genetic nurture”, because it implies that nurture – the totality of external factors after conception influenced or created by parents – may have a genetic component [1, 2]. Hence, genetic nurture arises from the influence of a heritable parental phenotype on the phenotype of their offspring, which is also known as vertical transmission [3]. Additional forms of indirect genetic effects can encompass other forms of cultural transmission, such as, horizontal transmission between individuals of the same generation, such as siblings [4, 5], and ecological inheritance, involving the transmission of inherited resources and conditions to descendants through niche construction [6].

Several designs can resolve direct genetic effects (the influence of genotype of the offspring on own phenotype) and indirect genetic effects (the above-mentioned genetic nurture). These include multi-generation twin-family designs that infer genetic effects from phenotypic resemblance among family members [7, 8], designs based on measured genetic information, e.g., polygenic scores (PGSs) [1, 2, 9, 10], or designs that combine these two [11]. Through PGS designs, robust evidence for genetic nurture effects in offspring educational attainment (EA) has been found [1, 2, 12].

The mechanism through which direct genetic effects (Mendelian inheritance) contribute to variation in human traits is complex, involving a cascade of transcription and translation of genetic information [13]. Here, we propose to examine DNA methylation as one possible mechanism through which both direct and indirect genetic effects may act. Epigenetic mechanisms such as DNA methylation modulate gene expression [14], but are themselves subject to both genetic [15, 16] and environmental influences [17]. Animal studies have shown that early life environmental exposures like diet, trauma, and social deprivation can induce epigenetic reprogramming in cells. For example, a classic study of rats found that the quality of maternal care early in life leads to epigenetic alterations that affect the behavior of offspring into adulthood [18]. Offspring raised by (non-biological) less-nurturing mothers exhibit increased anxiety as adults, which is attributed to enduring changes in DNA methylation in the brain. This study was based on cross-fostering experiments in which pups born to calm biological dams were raised by anxious adoptive dams, and vice versa, to separate the effect of the environment provided by the mother from genetic transmission [18].

In humans, it is more challenging to disentangle the influence of direct genetic transmission and indirect genetic effects that operate via the environment created by parents. Comprehensive reviews of childhood psychological adversity and DNA methylation indicated that childhood maltreatment and other adversities are associated with differential DNA methylation but called for further study of the impacts of childhood experiences and the effects of genetic transmission of parental psychopathology risk [19–21]. In the present study, we propose an approach to disentangle the influence of direct genetic transmission and indirect genetic effects that operate via the environment created on DNA methylation, by modelling the effects of parental PGSs and offspring PGSs on the offspring DNA methylation profile [9, 10]. Finding an effect of parental PGSs, in addition to the effect of the offspring’s own PGS, would support the hypothesis that genetic nurture acts through the offspring methylome. We obtained parental and offspring PGSs for six traits: schizophrenia, smoking initiation, EA, social deprivation, body mass index (BMI), and height in nuclear twin families. We focused on schizophrenia, EA, BMI, and height because they have high powered genome-wide association studies (GWAS). We focused on smoking due to its well-documented impact on DNA methylation as demonstrated by previous epigenome-wide association studies (EWAS) [22, 23]. Similarly, we examined social deprivation because of its strong theoretical basis suggesting mediation through epigenetic mechanisms [24]. EWAS have revealed widespread associations of DNA methylation and schizophrenia [25, 26], smoking [22, 23], and BMI [27, 28]. PGSs for these traits have been associated with DNA methylation [25, 29–31]. However, to date no studies have been undertaken to separate direct from indirect genetic effects on DNA methylation in offspring, which requires the PGSs of the offspring and the offspring’s parents.

In our study, the epigenetic data were available for 1528 young twins (51.5% boys, mean [*SD*] age = 10 [2.8] years). DNA samples from buccal cells for genotyping and polygenic scoring were available for the offspring and their parents. DNA methylation was measured by the Illumina Infinium EPIC array, which measures DNA methylation at approximately 850 K sites. To reduce multiple testing burden, we selected the top 10% most variable probes (72,889). We have shown in an independent sample of adult Dutch twins that the most variable probes are the most reliable across time, tissue, and platform [32]. DNA methylation levels in offspring were simultaneously regressed on offspring, maternal, and paternal PGSs by linear regression, while correcting for familial clustering.

## Materials and methods

### Study population and procedures

The collection of DNA samples in parents and their offspring was done at home from buccal swabs. Families were recruited from the Netherlands Twin Register (NTR) [33] and the majority took part in the ACTION Biomarker Study, which recruited twin pairs based on their (dis)similarity for childhood aggressive behavior [34, 35]. We first collected data in a pilot study to assess the suitability of the Infinium EPIC array for buccal-derived DNA samples [36] in 96 monozygotic (MZ) twins (47 complete pairs, 56.2% boys, mean [*SD*] (range) age at DNA collection 7.4 [2.4] (1–10) years). Next, 1141 twins (523 complete pairs, 52.6% boys, mean [*SD*] (range) age at DNA collection = 9.6 [1.9] (5.6–12.9) years) and their parents were included in the main ACTION Biomarker Study [37]. A third dataset included additional twins and siblings in the ACTION Biomarker Study, and unselected MZ twins and triplets from the NTR, totaling 291 children of 1 to 18 years of age (45.7% boys, mean [*SD*] age at DNA collection = 12.1 [4.2] years). DNA methylation data and offspring PGSs were available for all offspring, maternal PGSs were available for 92.1% and paternal PGSs for 81.1% of the families, with parental PGSs of both biological parents available for 78.3% of the families (Supplementary Table 1). All families were of European ancestry.

Parents gave written informed consent for their own and their offspring’s participation. The study was conducted according to the guidelines of the Declaration of Helsinki. The ACTION project was assessed by the Central Ethics Committee on Research Involving Human Subjects of the VU University Medical Center, Amsterdam (NTR 03-180, NTR 25th of May 2007, ACTION 2013/41 and 2014.252), an Institutional Review Board certified by the U.S. Office of Human Research Protections (IRB number IRB00002991 under Federal-wide Assurance FWA00017598).

### Infinium MethylationEPIC BeadChip

DNA methylation in offspring was assessed with the Infinium MethylationEPIC BeadChip Kit, following the manufacturer’s specification (Illumina, San Diego, CA, USA) [38]. A total of 500 ng of genomic DNA from buccal swabs were bisulfite-treated using the ZymoResearch EZ DNA Methylation kit (Zymo Research Corp, Irvine, CA, USA). Datasets 1 and 3 were generated at the Avera McKennan Hospital (Sioux Falls, SD, USA), and dataset 2 at the Human Genotyping Facility (HugeF) of ErasmusMC (Rotterdam, the Netherlands; http://www.glimdna.org/).

As previously described [36, 39], quality control (QC) and normalization of the methylation data were performed with the pipelines developed by the Biobank-based Integrative Omics Study (BIOS) consortium [40]. The probe β-values represent the ratio of the methylated signal intensity to total signal intensity (methylated plus unmethylated) at a given CpG site and can range from 0 (unmethylated) to 1 (fully methylated). Only samples that passed all five quality criteria of MethylAid were retained [41]. In addition, these samples were characterized by correct genetic relationships among the participants (omicsPrint) [42] and the absence of sex mismatches (DNAmArray and meffil) [43]. Functional normalization was performed with the dataset-specific optimum number of principal components. Methylation probes were coded as missing, if they had an intensity value of zero, bead count <3, or detection *p*-value > 0.01. A probe was excluded if it overlapped with a SNP or Insertion/Deletion (INDEL), mapped to multiple locations in the genome, or had a success rate <0.95 across all samples. Sample QC, probe filtering, normalization, and imputation of missing values were done within the three datasets separately. Of 865,859 sites on the array, 789,888 sites, 787,711 sites, and 734,807 sites were retained after QC in datasets 1, 2, and 3, respectively, and we retained the 728,899 overlapping sites. Cellular proportions were predicted in epithelial tissues based on the cell-type deconvolution algorithm Hierarchical Epigenetic Dissection of Intra-Sample-Heterogeneity (HepiDISH) with the reduced partial correlation [44]. After QC, we removed all cross-reactive probes, probes in SNPs or on the X or Y chromosome and then imputed missing methylation β-values (up to 5%; probes with higher missingness were excluded) with the imputePCA() function (missMDA R in the BIOS pipeline). Next, we removed methylation outliers (range: 291–505) that exceeded three times the interquartile range (ewaff R) [45] in each dataset. We then combined the methylation β-values from all three imputed datasets and obtained residual methylation levels by regressing the effects of sex, age, percentages of epithelial and natural killer cells, EPIC array row, and bisulfite sample plate. In the combined dataset of imputed and residualized DNA methylation sites, we calculated the top 10% most variable methylation sites (72,889) to retain for further analysis (Supplementary Fig. 1, Supplementary Table 2).

### Genotyping and calculation of polygenic scores

Genotyping was done according to the manufacturer’s protocols in 3124 NTR samples on multiple genotyping platforms. Sample and SNP QC was performed per genotyping platform (build 37) for all individuals in the NTR. Genotype data were imputed to 1000 genomes phase 3 (v.5) [46] and a combined HRC 1.1 (EGA version) [47, 48] and GONL (v.4) [49] reference panel. The first was the basis for calculation of genetic principal components (PCs) and the second for calculation of PGSs. More details on QC and imputation are included in Supplementary Note 1 and on calculation of genetic PCs in Supplementary Note 2.

Parental and offspring PGSs for schizophrenia [50], smoking initiation [51], educational attainment [52], social deprivation [53], BMI [54], and height [54] were calculated based on GWASs, that all omitted NTR from the discovery (meta-)analysis. Before generating the PGSs, QC steps involved removing SNPs with Hardy-Weinberg equilibrium (HWE) p-values below 0.00001, Mendelian error rates above 1%, genotype call rate below 98%, effect allele frequencies below 0.01 and above 0.99, imputation info below 0.10 and SNPs showing over 2% differences in allele frequencies between genotyping platforms. We retained 7,086,504 (schizophrenia), 6,982,922 (smoking initiation), 7,118,026 (EA), 6,997,217 (social deprivation), 2,220,360 (BMI), and 2,206,961 (height) SNPs.

LD-weighted betas were calculated from the processed summary statistics in the LDpred package (v.0.9) to correct for the effects of LD, and to maximize predictive accuracy of the PGS [55]. We randomly selected 2500 individuals unrelated to the 2nd degree from the NTR as a reference population to obtain LD patterns in an NTR genotype subset of well imputed variants. Weights to obtain the PGSs in PLINK2 (–score) [56] were calculated with an LD pruning window of 250 KB and the infinitesimal prior (LDpred-inf). We calculated the offspring, maternal, and paternal PGS for the 6 traits (18 PGSs total). Two dummy coded genotyping platforms and the first 10 genetic PCs were regressed on the PGSs. The standardized residuals (unit variance and zero means) were analyzed. If maternal or paternal polygenic scores were missing, we applied mean imputation (i.e., imputed the parental PGS with zero) [2] (*N*_*dataset1*_ = 31; *N*_*dataset2*_ = 213; *N*_*dataset3*_ = 87).

### Analyses

As shown in Okbay et al. [9] (see also Supplementary Note 3), models that include the PGSs from offspring and two parents provide estimates of direct and indirect genetic effects, and allow for differences in maternal and paternal indirect genetic effects [9, 10].

Direct and indirect genetic effects for a single trait can be estimated by fitting the following regression model:

1 $${{{{\rm{Y}}}}}_{{{{\rm{ij}}}}}={{{\rm{\mu }}}}+{{{{\rm{\delta }}}}{{{\rm{PGS}}}}}_{{{{\rm{ij}}}}}+{{{{\rm{\alpha }}}}}_{{{{\rm{m}}}}}{{{{\rm{PGS}}}}}_{{{{\rm{m}}}}({{{\rm{i}}}})}+{{{{\rm{\alpha }}}}}_{{{{\rm{p}}}}}{{{{\rm{PGS}}}}}_{{{{\rm{p}}}}({{{\rm{i}}}})}+{{{{\rm{\varepsilon }}}}}_{{{{\rm{ij}}}}},$$

where Y_ij_ is the residualized methylation probe β-value, and i and j index family and individual; μ is the intercept, δ is the direct effect of the offspring PGS; α_m_ captures the maternal indirect genetic effects; α_p_ captures the paternal indirect genetic effects; PGS_m(i)_ and PGS_p(i)_ are the PGSs of the mother and father in family i; and ε_ij_ is a residual term.

We extended Eq. 1 by including maternal, paternal, and offspring PGS for multiple traits. This allows for the unbiased estimation of the direct and indirect genetic effects for a given complex trait, while accounting for direct and indirect effects on all other traits in the model.

The direct and indirect genetic effects for multiple traits are modelled as follows:

2 $${{{{\rm{Y}}}}}_{{{{\rm{ij}}}}}=	 \, {{{\rm{\mu }}}}+{{{\rm{\delta }}}}{{PGS}}_{{ij}}^{{trait} \, 1}+{{{{\rm{\alpha }}}}}_{{{{\rm{m}}}}}{{PGS}}_{m(i)}^{{trait} \, 1}+{{{{\rm{\alpha }}}}}_{{{{\rm{p}}}}}{{PGS}}_{p(i)}^{{trait} \, 1}+\ldots +{{{\rm{\delta }}}}{{PGS}}_{{ij}}^{{trait} \, N} \\ 	+{{{{\rm{\alpha }}}}}_{{{{\rm{m}}}}}{{PGS}}_{m(i)}^{{trait} \, N} +{{{{\rm{\alpha }}}}}_{{{{\rm{p}}}}}{{PGS}}_{p(i)}^{{trait} \, N}+{{{{\rm{\beta }}}}}_{1}{{{{\rm{Cov}}}}}_{{{{\rm{ij}}}}}+{{{{\rm{\beta }}}}}_{2}{{{{\rm{Cov}}}}}_{{{{\rm{ij}}}}}+{{{{\rm{\varepsilon }}}}}_{{{{\rm{ij}}}}},$$

where subscript trait 1 through trait N indicates the trait for which the standardized residual polygenetic score was calculated and Cov_ij_ and Cov_*i*j_ indicate the DNA methylation dataset.

We tested the association between the 72,889 CpGs and the offspring, maternal, and paternal PGSs for the six traits simultaneously Eq. 2, i.e., 18 predictors, using Generalized Estimation Equation (GEE) regression modeling to correct the standard errors for family clustering of individuals [57]. All analyses were carried out in R (version 4.2.2) [58] in the R package ‘gee’ (version 4.13–26) [59]. A Bonferroni correction was applied for the number of independent DNA methylation probes tested (*α* = 0.05/N independent variables), following the procedure outlined by Nyholt [60], where the number of independent tests is determined by Matrix Spectral Decomposition (MSD) of the imputed CpGs. The correlated CpGs could be reduced to 1850 independent linear combinations of the probes and we set the significance level to (0.05/1850) = 2.7 × 10^−5^. When analyzing the 72,889 CpGs, the regression model for 192 CpGs did not converge. We present the results for the remaining 72,697 successful analyses.

Significant CpGs from these analyses were followed up in four analyses. First, we queried the EWAS Atlas database to identify with which traits these CpGs had been previously associated [61]. Second, we queried the GoDMC database (http://mqtldb.godmc.org.uk/) for associated blood *cis* and *trans* methylation quantitative trait loci (mQTLs) for these CpGs [15] and examined if the identified mQTLs were mapped to genome-wide significant (*p* < 5 × 10^−8^) SNPs for the respective traits. Third, we investigated whether the DNA methylation levels in buccal cells and prefrontal cortex were significantly correlated (Spearman rank correlations at False Discovery Rate (FDR) *q* < 0.05) in a published dataset of matched post-mortem samples (*N* = 120) [62]. Fourth, we performed a look-up of the genes annotated to the significantly associated CpGs to investigate whether they are protein expressing in the human dorsal lateral prefrontal cortex and if so, whether these proteins are differentially regulated in schizophrenia [63]. Last, we performed trait enrichment analyses of the top 100 most significant sites identified in the mega-analysis for each PGS and complex trait in the EWAS Atlas.

## Results

**Fig. 1:**
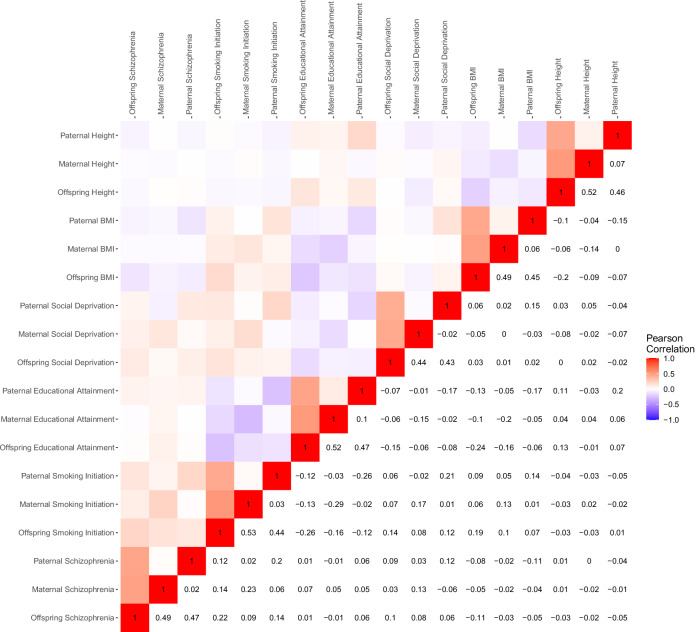
Pearson correlations among the offspring, maternal, and paternal polygenic scores for schizophrenia, smoking initiation, educational attainment, social deprivation, body mass index BMI, and height.

Participant characteristics are described in Table 1. We analyzed data from 1528 offspring and their parents (51.5% boys, mean [SD] age = 10 [2.8] years) to test the simultaneous association of offspring, maternal, and paternal PGSs for six complex traits with 72,697 buccal DNA methylation sites in offspring. The correlations between PGSs of different traits ranged from −0.29 and 0.23 (mean = −0.004, median = −0.01), with the strongest correlation observed between the maternal PGSs of smoking initiation and EA (Fig. 1). We accounted for these correlations by fitting the PGSs of all traits simultaneously in one model. The correlations between the offspring and parental PGSs for the same trait were approximately 0.5 as expected (range = 0.43–0.53, mean = 0.48, median = 0.47), while correlations between parental PGSs for the same trait were on average 0.04 (median = 0.04, range = −0.02–0.10).

**Table 1:** Demographics of the individuals included across the three buccal DNA methylation datasets in this study.

	MZ	DZ	Triplets	Siblings	Total^a^
*N* (complete twin pairs or trios)	1170 (538)	209 (99)	34 (9)	115	1528
Mean (*SD*) [range] age	9.8 (2.7) [1–18]	9.9 (1.7) [5.7–13]	7.7 (5.6) [2–17]	11.9 (2.6) [5–17]	10 (2.8) [1–18]
*N* (%) males	614 (52.5%)	101 (48.3%)	18 (52.9%)	54 (47%)	787 (51.5%)

*MZ* monozygotic twins, *DZ* dizygotic twins.

^a^Parental polygenic scores for one or both parents were missing for 331 children, these parental PGS were mean imputed.

**Fig. 2:**
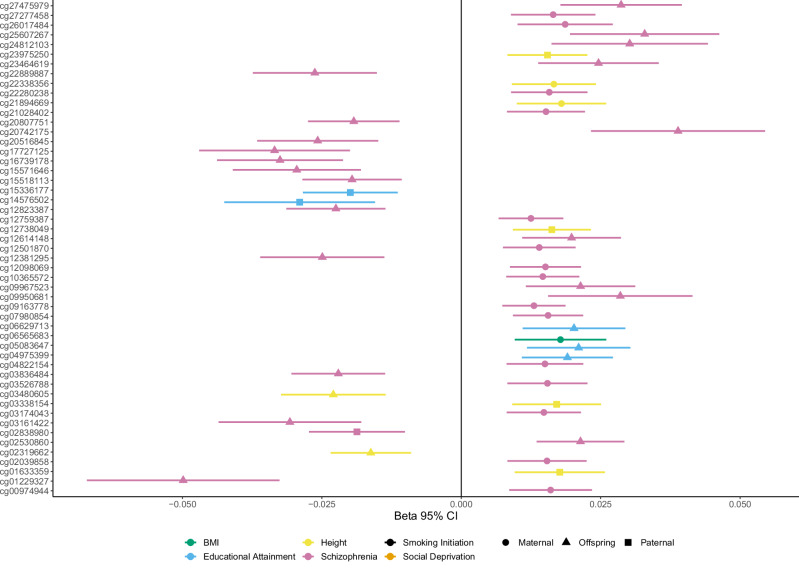
Buccal CpGs significantly associated (alpha = 2.7 × 10^−5^) with maternal (○), paternal (□), or offspring ($\triangle$) polygenic scores for schizophrenia, educational attainment, body mass index (BMI), and height.

We observed independent significant associations between offspring methylation sites and maternal PGSs for schizophrenia (15), height (2), and BMI (1), and paternal PGSs for schizophrenia (1), EA (2), and height (4), (Fig. 2, Table 2), demonstrating a mechanism for indirect effects from parental phenotype to the offspring methylome. The own offspring PGSs for schizophrenia, EA, and height showed associations with 21, 3, and 2 offspring methylation sites, respectively. None of the CpGs associated with maternal, paternal, or offspring PGSs overlapped. Thus, in total, 25 and 26 significant CpG sites (*p* < 2.7 × 10^−5^, Bonferroni correction for 1850 tests) were associated with indirect and direct genetic effects across the six complex traits, respectively (Supplementary Figs 2–7, Supplementary Table 3).

**Table 2:** Buccal CpGs significantly associated (alpha = 2.7 × 10^−5^) with maternal, paternal, or offspring polygenic scores for schizophrenia, educational attainment (EA), body mass index (BMI), and height.

PGS	CpG	CHR:BP^a^	Gene^b^	mQTLs	SNPs	Complex traits
Maternal Schizophrenia	cg00974944	3:61649924	*PTPRG*			
Maternal Schizophrenia	cg02039858	14:65828769				
Maternal Schizophrenia	cg03174043	2:20548412		185		fractional exhaled nitric oxide
Maternal Schizophrenia	cg03526788	8:130899478	*FAM49B*			
Maternal Schizophrenia	cg04822154	8:124692680				
Maternal Schizophrenia	cg07980854	20:57586338		634		breast cancer; type 2 diabetes; atopy; psoriasis
Maternal Schizophrenia	cg09163778	6:31125417	*TCF19; CCHCR1*			
Maternal Schizophrenia	cg10365572	5:141245930	*PCDH1*			
Maternal Schizophrenia	cg12098069	13:42537172	*VWA8-AS1*			
Maternal Schizophrenia	cg12501870	8:81803721		182		
Maternal Schizophrenia	cg12759387	1:27849177		177		systemic lupus erythematosus; polychlorinated biphenyls exposure
Maternal Schizophrenia	cg21028402	6:113932902		297		helicobacter pylori infection; oral squamous cell carcinoma
Maternal Schizophrenia	cg22280238	17:79251323	*SLC38A10*			
Maternal Schizophrenia	cg26017484	1:3322309	*PRDM16*			adenoma
Maternal Schizophrenia	cg27277458	10:63597194				
Paternal Schizophrenia	cg02838980	16:10402487				
Offspring Schizophrenia	cg01229327	19:54311998	*NLRP12*			
Offspring Schizophrenia	cg02530860	8:144371537		646		gender; sperm viability; assisted reproduction technology; infant sex; sexually dimorphic
Offspring Schizophrenia	cg03161422	4:175256045				
Offspring Schizophrenia	cg03836484	10:25349182	*ENKUR*			
Offspring Schizophrenia	cg09950681	11:393392	*PKP3*			
Offspring Schizophrenia	cg09967523	18:18943072	*GREB1L*			
Offspring Schizophrenia	cg12381295	20:3880297	*PANK2*			gingivo-buccal oral squamous cell carcinoma (OSCC-GB)
Offspring Schizophrenia	cg12614148	1:118325026		98		
Offspring Schizophrenia	cg12823387	1:214614656	*PTPN14*			
Offspring Schizophrenia	cg15518113	1:167400121	*CD247*	105		Air pollution (NO2); adrenocortical carcinoma; smoking; multiple sclerosis;
Offspring Schizophrenia	cg15571646	2:238767351	*RAMP1*			
Offspring Schizophrenia	cg16739178	16:85470674		50		papillary thyroid carcinoma; aging; BMI; Crohn’s disease; Behcet’s disease; maternal hypertensive disorders in pregnancy; gestational age; blood C-reactive protein level; inflammatory bowel disease
Offspring Schizophrenia	cg17727125	8:38441156				
Offspring Schizophrenia	cg20516845	21:43823604	*UBASH3A*	140		
Offspring Schizophrenia	cg20742175	20:57267357	*NPEPL1;* *STX16-NPEPL1*			high risk cutaneous squamous cell carcinoma (cSCC)
Offspring Schizophrenia	cg20807751	9:84025287				
Offspring Schizophrenia	cg22889887	11:113433931		6		
Offspring Schizophrenia	cg23464619	13:95513506		50		hepatocellular carcinoma; colorectal laterally spreading tumor; inflammatory bowel disease; ulcerative colitis
Offspring Schizophrenia	cg24812103	8:101735630	*PABPC1*	244		prenatal arsenic exposure; gestational diabetes mellitus; inflammatory bowel disease
Offspring Schizophrenia	cg25607267	12:122362708	*WDR66*			
Offspring Schizophrenia	cg27475979	16:89471039	*ANKRD11*			
Paternal EA	cg14576502	8:102962279	*NCALD*			recurrent adult-type IDH-mutant gliomas
Paternal EA	cg15336177	1:99369228	*LPPR5*			
Offspring EA	cg04975399	1:67822225	*IL12RB2*			fractional exhaled nitric oxide
Offspring EA	cg05083647	1:204897364	*NFASC*			
Offspring EA	cg06629713	2:60580559		43		primary Sjogren’s Syndrome; obesity; Type 2 diabetes; asthma; atopy; Crohn’s disease; fractional exhaled nitric oxide; hepatitis B virus-related liver disease progression
Maternal BMI	cg06565683	20:46817989				
Maternal Height	cg21894669	2:165379442	*GRB14*			
Maternal Height	cg22338356	8:48739161	*PRKDC*			Down Syndrome; Fetal alcohol spectrum disorder
Paternal Height	cg01633359	5:33439359		164	56	
Paternal Height	cg03338154	6:41121714	TREML1			
Paternal Height	cg12738049	15:76509692	ETFA			
Paternal Height	cg23975250	22:44757380				sexually dimorphic
Offspring Height	cg02319662	4:108127694				
Offspring Height	cg03480605	7:151038446	NUB1	216	17	maternal smoking; obesity; oral squamous cell carcinoma

^a^CHR:BP = Chromosome base pair location in genome build 37.

^b^Gene = NCBI gene summaries and Ensebl links are added to Supplementary Table 8. mQTLs = number of blood *cis* methylation quantitative trait loci (mQTLs) [15] associated with the CpGs. SNPs = number of genome-wide significant (*p* < 5 × 10^−8^) SNPs overlapping with blood *cis* mQTLs for the CpGs. Complex traits = complex traits previously associated with the CpGs in the EWAS Atlas [61].

The query of the EWAS Atlas database [61] for the 37 CpGs associated with direct or indirect genetic effects for schizophrenia showed that 12 CpGs (5 associated with the maternal and 7 with the offspring PGS) had known trait associations, though not with schizophrenia or other mental health traits (Table 2). Similarly, 3 out of the 5 CpGs associated with paternal (2) or offspring (3) PGSs for EA and 3 out of the 8 CpGs associated with maternal (2), paternal (4), or offspring (2) PGSs for height were included in the EWAS Atlas but showed no previous associations with the respective or a related complex trait. Trait enrichment analyses for the top 100 CpGs associated with offspring and parental PGSs for all six complex traits showed enrichment for 5 to 18 traits per PGS (mean = 10.2, median = 10) with on average 2.2 CpGs associated per trait (range = 1–7, median = 2, Supplementary Table 4). Notably, many of the enriched traits are phenotypically associated with the relevant PGSs, such as enrichment for bipolar and major depressive disorder and maternal hypertensive disorders in pregnancy for CpGs associated with maternal indirect genetic effects for schizophrenia and household socioeconomic status in childhood for CpGs with direct and indirect genetic effects for schizophrenia.

A query of the GoDMC blood mQTL database [15] with the 51 significant CpGs identified 3237 *cis* mQTLs for 16 CpGs (mean = 202.3 mQTLs per associated CpG, median = 170.5) associated with schizophrenia (13 CpGs), EA (1), and height (2), with roughly half (1639/3237) of the mQTLs associated with indirect genetic effect CpGs (Table 2, Supplementary Table 5). Of the 164 mQTLs associated with cg01633359 (paternal PGS for height) and 216 of the mQTLs associated with cg03480605 (offspring PGS for height) 56 and 17, respectively, had previously been associated with height at genome-wide significant levels (*p* < 5 × 10^−8^, Table 2, Supplementary Fig. 8, Supplementary Table 6) [54]. None of the mQTLs for the schizophrenia and EA associated CpGs were previously found to be significantly associated with their respective traits at genome-wide significant levels [50, 52].

We looked at CpGs significantly associated with direct or indirect genetic effects in a published dataset (*N* = 120) of correlations between DNA methylation levels in buccal cells and the prefrontal cortex [62]. One CpG associated with the maternal PGS for schizophrenia (cg09163778, *r* = 0.31, *p* = 5.86 × 10^−4^), 2 CpGs associated with the offspring PGS for schizophrenia (cg02530860, *r* = 0.67, *p* = 7.51 × 10^−17^, cg01229327, *r* = 0.49, *p* = 1.91 × 10^−8^), and one CpG associated with the paternal PGS for height (cg01633359, *r* = 0.29, *p* = 1.44 × 10^−3^) showed significantly (FDR *q* < 0.05) correlated DNA methylation levels between buccal cells and the prefrontal cortex (Supplementary Table 7). Because of the relatively large number of CpGs associated with schizophrenia PGSs, we performed further follow-up analyses on the differential regulation in schizophrenia of the proteins encoded by the 29 genes annotated to these CpGs. Of these genes, 9 genes had protein expression detected in the dorsal lateral prefrontal cortex (dlPFC) and 2 of those (*PRKDC* and *NCALD*) were significantly differentially regulated in schizophrenia (Supplementary Table 8). Neither gene was annotated to a CpG associated with the offspring or parental PGS for schizophrenia, rather *PRKDC* was annotated to cg22338356 (chr8:48739161) which was associated with the maternal PGS for height, and *NCALD* was annotated to cg14576502 (chr8:102962279) which was associated with the paternal PGS for EA.

## Discussion

We investigated direct and indirect genetic influences on offspring DNA methylation for schizophrenia, smoking initiation, EA, social deprivation, BMI, and height in a cohort of 1528 children and their parents. We identified 37 CpGs significantly associated with schizophrenia, 5 with EA, 1 with BMI, and 8 with height. None of the identified CpGs overlapped between polygenic scores. We found the strongest genetic nurture effect for schizophrenia. Of the schizophrenia associated CpGs, 43% (16 CpGs, 93.7% maternal) showed an association with parental PGSs. Overall, the maternal genetic nurture effect for schizophrenia (15 CpGs) on offspring DNA methylation represented 83.3% of all maternal genetic nurture effects (18 CpGs) and 58% of all genetic nurture effects (25 CpGs).

We identified 25 CpGs associated with parental PGSs. These results indicate that one mechanism for genetic nurture is via the offspring methylome, though the relation of the identified CpGs with offspring outcomes remains to be determined. Of these, 18 CpGs were specific to associations with maternal PGSs, and 7 were specific to paternal PGSs, suggesting that genetically driven paternal behaviors have a smaller impact on offspring DNA methylation. The larger number of CpGs associated with maternal PGSs for schizophrenia (15/16 CpGs) is consistent with a stronger influence of genetic nurture effects on offspring DNA methylation in utero. This aligns with the “Developmental Origins of Health and Disease” (DOHad) hypothesis, that prenatal environmental factors have long-term effects through epigenetic mechanisms [64]. Unfavorable intrauterine conditions leading to poor fetal growth are particularly relevant to the DOHaD hypothesis [65, 66]. Observational studies indicate that the intrauterine environment is influenced by maternal height and weight, and both direct and maternal indirect genetic effects affect offspring birth length, weight, and gestational age [67, 68]. The CpGs associated with maternal PGSs for height (2 CpGs) and BMI (1 CpG) may be involved in these processes. However, recent research suggests that the impact of intrauterine growth restriction on complex disease risk is limited, and that non-transmitted maternal genetic factors for birth weight do not contribute to offspring disease risk beyond genetic pleiotropy with offspring genetic factors [69]. This aligns with our finding that the largest number of findings for non-transmitted maternal genetic factors is not for body size but for schizophrenia. Most CpGs associated with indirect genetic effects were observed for maternal PGSs, particularly for schizophrenia. This observation may result from both pre-and postnatal exposures, including rearing environment and maternal parenting behaviors. One direction for further research is to examine DNA methylation in neonatal samples to better isolate prenatal from postnatal effects.

Several of the CpG sites associated with the offspring and parental PGSs for schizophrenia annotate to genes implicated in neurodevelopmental and immune processes (Table 2, Supplementary Table 8), consistent with the pathophysiology of schizophrenia [70, 71]. For instance, *PTPRG* (cg00974944) and *PCDH1* (cg10365572), both associated with the maternal schizophrenia PGS, are involved in cell growth regulation and neural adhesion, respectively, supporting the role of dysregulated synaptic processes in schizophrenia risk [50]. Similarly, *CCHCR1* (cg09163778, associated with maternal schizophrenia PGS), located within the major histocompatibility complex (MHC) region, is of particular interest due to the MHC’s established role in immune regulation and its prior associations with schizophrenia [72].

For EA, several genes associated with CpG sites suggest mechanisms related to neuronal signaling and synaptic plasticity (Table 2, Supplementary Table 8). For example, *NCALD* (cg14576502, associated with the paternal PGS for EA), a gene encoding a calcium-binding protein involved in G-protein-coupled receptor signal transduction, may influence cognitive functions through its regulatory role in calcium signaling, a key process in synaptic activity and plasticity [73]. We also observed significant regulation of *NCALD* protein expression in the dlPFC in schizophrenia.

For height, several CpG sites annotate to genes related to growth, metabolism, and cellular signaling (Table 2, Supplementary Table 8). *GRB14* (cg21894669, associated with maternal PGS for height), encodes a growth factor receptor-binding protein that regulates insulin receptor signaling. By modulating receptor tyrosine kinase activity, particularly in insulin pathways, it likely influences growth and metabolism. Additionally, *PRKDC* (cg22338356, associated with the maternal PGS for height), one of the two genes with significantly regulated protein expression in the dlPFC in schizophrenia, is highly expressed in immune cells and mutations have been linked to autoimmune diseases and increased immune response [74]. This connection between immune system regulation, schizophrenia, and height reflects previous findings from GWAS studies [75].

We did not model a relation of the identified CpGs with offspring outcomes, but explored the literature to determine whether the significant CpGs for each trait had been associated with the PGS for that trait and found that none of the CpGs significantly associated with direct or indirect genetic effects had been associated with polygenic scores for schizophrenia or BMI [25, 29–31]. These previous studies all comprised adults, whereas our study focused on children. The previous studies on schizophrenia mainly comprised adult patient populations focused on DNA methylation in blood or post-mortem brain samples (*N* range = 88–847). DNA methylation is often tissue specific and despite identifying blood *cis* mQTLs for approximately one-third of the CpGs associated with direct and indirect genetic effects, some *cis* mQTLs are not found in blood. Aside from tissue-specificity, the lack over overlap between CpGs associated in buccal and blood *cis* mQTLs might reflect differences in DNA methylation arrays. Previously, we found that 46.3% of buccal mQTLs as measured on the Illumina EPIC array overlap with blood mQTLs as measured on the Illumina 450 K array when 15,897 of 33,749 EPIC sites were present on the 450 K [36]. As blood and buccal *cis* mQTLs have a high (71%) concordance, we expect to have identified most *cis* mQTLs [76]. None of the identified CpGs were associated with *trans* mQTLs. Three CpGs associated with schizophrenia PGSs (1 maternal, 2 offspring) and one CpG associated with the paternal PGS for height showed correlated methylation levels between buccal cells and prefrontal cortex brain samples [62]. Future studies should investigate direct and indirect genetic effects in multiple tissues relevant to health outcomes to better understand tissue-specific versus tissue-agnostic DNA methylation signatures.

This study is the first to test if indirect genetic effects influence DNA methylation in offspring. It also explored the direct genetic effects on DNA methylation in children. We analyzed data from 1528 children and their parents. Most children were related to at least one other person in the sample, such as their co-twin and thus the effective sample size is smaller than 1528. Given the sample size, we took three measures to reduce the multiple testing burden. First, we focused on the top 10% most variable CpGs, which have been shown to be the most reliable [32]. Second, we adjusted our multiple testing threshold based on the number of independent CpGs, considering the high correlations between them. Third, our approach of modeling multiple complex traits simultaneously allowed us to avoid correcting for the number of traits investigated, which would have been necessary in a separate modeling strategy. By limiting our investigation to highly variable CpGs, we cannot draw conclusions on less variable CpGs or CpGs with strong temporal patterns. Overall, our application highlights its potential to investigate epigenetic mechanisms of indirect genetic effects and encourages testing in large family cohorts, across different tissues, and populations.

In summary, we found support for the hypothesis that indirect genetic effects are associated with DNA methylation in offspring. By simultaneously modelling multiple complex traits, we found robust associations of indirect genetic effects on offspring DNA methylation for schizophrenia, EA, BMI, and height, but not smoking initiation and social deprivation. Most of these associations were discovered in relation to maternal PGSs, consistent with the interpretation that the prenatal environment influences the DNA methylation of offspring during embryonic development or reflect a stronger maternal influence on the offspring’s environment during the pre- and perinatal period and early childhood.

## Supplementary information

Supplementary Material

Supplementary Tables

## Data Availability

The standardized protocol for large scale collection of buccal-cell (and urine) samples in the home situation as developed for the ACTION Biomarker Study in children is available at 10.17504/protocols.io.eq2ly7qkwlx9/v1 and at https://www.action-euproject.eu/content/data-protocols. The data of the Netherlands Twin Register (NTR) may be accessed, upon approval of the data access committee, through the NTR (https://ntr-data-request.psy.vu.nl/).

## Code availability

The pipeline for DNA methylation–array analysis developed by the Biobank-based Integrative Omics Study (BIOS) consortium is available at https://molepi.github.io/DNAmArray_workflow/ (10.5281/zenodo.3355292). All software used to perform the analyses are available online. All scripts used to run the analyses (empirical and simulated) are available in our GitHub repository https://github.com/FionaAHagenbeek/MethylationPGS (https://zenodo.org/records/14960968).
